# The First Mitochondrial Genome of Family Xylococcidae (Hemiptera, Coccomorpha) and Its Phylogenetic Implications

**DOI:** 10.3390/genes16050594

**Published:** 2025-05-17

**Authors:** Xiaxia Zhao, Yuang Li, Han Xu, Sanan Wu

**Affiliations:** The Key Laboratory for Silviculture and Conservation of Ministry of Education, Beijing Forestry University, Beijing 100083, China; zx1599805604@163.com (X.Z.); liyuang1124@163.com (Y.L.); sananwu@bjfu.edu.cn (S.W.)

**Keywords:** coccomorpha, gene rearrangements, mitogenome, molecular phylogeny

## Abstract

Background: The mitochondrial genome has been used for understanding higher-level phylogenetic relationships within Coccomorpha. we sequenced and analyzed the first mitochondrial genome of *Xylococcus castanopsis* Wu & Huang, 2017 to elucidate its genomic features and phylogenetic position. Methods: The complete mitogenome was assembled using NOVOPlasty and annotated with MITOS. We analyzed genome organization, codon usage, and tRNA structures. Phylogenetic relationships were reconstructed using 13 protein-coding genes from 19 scale insect species with Bayesian Inference and Maximum Likelihood methods. Result: The mitochondrial genome is 16,363 bp in size and contains the typical 37 mitochondrial genes, with an A + T content of 89.2%. All protein-coding genes start with the ATN and end with TAA/TAG or a single T- residue. Sixteen tRNAs exhibit the typical cloverleaf structure, while the remaining six lack either the dihydrouridine (DHU) or TΨC (T) arm. Gene rearrangements occur only in individual tRNAs and transpositions between the gene clusters *trnS2-ND1* and *trnL1-rrnL-trnV-rrnS*. Phylogenetic analysis consistently place Xylococcidae as a sister group to all scale insects except Matsucoccidae. Conclusions: This study provides the first complete mitogenome for Xylococcidae, revealing characteristic gene rearrangements. Phylogenetic reconstruction resolves the phylogenetic position of Xylococcidae as a distinct lineage sister to all scale insects except Matsucoccidae, providing critical evolutionary insights.

## 1. Introduction

Scale insects (Hemiptera: Coccomorpha) are small and live hidden, causing plant weakening and pathogen transmission through feeding on phloem sap, which in severe cases can even lead to plant death [1]. Currently, there are more than 8500 described species in 1238 genera, belonging to 35 extant and 21 extinct families [2]. They were divided into two informal groups: archaeococcoids and neococcoids. The archaeococcoids typically feature abdominal spiracles, at least in females. However, neococcoids lack the abdominal spiracles and multifaceted eyes [3,4].

The taxonomic status of the subfamily Xylococcinae has undergone multiple revisions. It was considered a part of the family Margarodidae sensu lato by various authors, such as Morrison [5], who recognized it as the tribe Xylococcini. Zahradnik elevated Xylococcinae to family rank [6], while Koteja restricted Xylococcidae to two genera [7]: *Xylococcus* and *Xylococculus*. Therefore, the relationships within Margarodidae sensu lato, particularly regarding the systematic position of Xylococcidae, remain unresolved. These conflicting classifications demonstrate the persistent difficulties in establishing a satisfactory system for Coccomorpha. For consistency, we follow Koteja’s [7,8] classification, which treats Xylococcidae as a distinct family.

The family Xylococcidae currently includes four genera: *Baisococcus* Koteja, 1989; *Mesophthirus* Gao, Shih, Rasnitsyn & Ren, 2019; *Xylococculus* Morrison, 1927; and *Xylococcus* Lôw, 1882. However, only seven extant species remain within *Xylococcus* (*Xylococcus castanopsis* Wu & Huang, 2017; *Xylococcus filiferus* Löw, 1882; *Xylococcus japonicus* Oguma, 1926; *Xylococcus quercicola* Danzig, 1980); *Xylococculus* (*Xylococculus betulae* (Pergande, 1898); *Xylococculus macrocarpae* (Coleman, 1908); and *Xylococculus quercus* (Ehrhorn, 1900), while the other two genera are extinct [2]. In China, only *X. castanopsis* has been recorded [9]. Remaining extant species are distributed in North America, Northeast Asia and Europe [2,10].

Mitochondria, essential organelles present in nearly all eukaryotic cells, are crucial for energy production [11]. Mitochondrial genomes of insects are now widely used in studies on species identification, population genetic structure, biogeography, and phylogenetics [12,13]. However, the current research on mitochondrial genomes of scale insects remains relatively sparse. Only a handful of cocciologists have published studies on their mitochondrial genomes, with the studies primarily focusing on their structure and characteristics and exploring phylogenetic relationships based on existing mitochondrial genomes [14,15,16,17]. Despite the high diversity of scale insects, only mitogenomes of 24 species have been recorded in NCBI.

Whether based on phylogenetic trees reconstructed from the morphological characteristics of adult males or those combining molecular data with morphological traits, the position of Xylococcidae remains unresolved, and its mitochondrial genome has yet to be documented. In this paper, the mitogenome of *X. castanopsis* was sequenced and analyzed in detail. We then performed a phylogeny analysis of Coccomorpha mitogenomes and tried to explore the systematic position of Xylococcidae. This contributes to a better understanding of the phylogenetic relationships within Coccomorpha.

## 2. Materials and Methods

### 2.1. Sampling, Genomic DNA Extraction and Sequencing

The sample for DNA extraction was collected from the bark of *Castanea henryi* (Skan) Rehd. et Wils. (Fagaceae) from Guangdong Province, China, and was preserved in 95% ethanol under −20 °C at the Beijing Forestry University, Beijing, China (BFUC). The total genomic DNA was extracted using the TIANamp Genomic DNA Kit, following the manufacturer’s protocol. Voucher specimens are deposited at Beijing Forestry University, Beijing, China (BFUC). The *COX1* sequences were amplified using the primers C1-1554F and C1-2342R [18] and sequenced via the Sanger method on an ABI 3730xl sequencer to provide seed sequences for subsequent mitochondrial genome assembly. We sequenced the genome of *X. castanopsis* using next-generation sequencing on the Illuminia Hiseq 2500 platform at Berry Genomics, Beijing, China. The sequencing achieved a depth of 6× with 150 bp paired-end reads. A single-species Illumina TruSeq library was prepared from total genomic DNA, featuring an average insert size of 150 bp. The sequence data of phylogenetic analysis were obtained from the National Center for Biotechnology Information at https://www.ncbi.nlm.nih.gov (refer to Table 1).

### 2.2. Mitogenome Assembly and Annotation

Following the removal of adapters and low-quality reads, we obtained approximately 12 GB of clean data and assembled it for reference-guided assembly using NOVOPlasty v4.3.3 [19], with the mitochondrial genome of *Matsucoccus matsumurae* as the reference and the *COX1* sequence of *X. castanopsis* serving as a bait. This process successfully yielded a mitogenome of 16,363 bp. Then, annotated using the MITOS v2.1.9 on the Galaxy platform, the accurate boundaries of protein-coding genes (PCGs) were confirmed by comparison with other scale insects using MEGA 7.0.26 [20,21]. Six tRNA genes (*trnA*, *trnQ*, *trnL1*, *trnS2*, *trnW*, and *trnV*) were not identified by MITOS v2.1.9; however, they were successfully detected using the manual method and ARWEN v1.2 [22]. The *rrnS* was detected by MITOS v2.1.9, and the *rrnL* was determined by the upstream and downstream of tRNAs and alignment with the other scale insects.

### 2.3. Bioinformatic Analysis

Base composition, codon usage of protein-coding genes (PCGs), and relative synonymous codon usage (RSCU) values were calculated using MEGA v7.0.26. Figures were then generated using the ggplot2 package in R v4.3.2 [23,24]. Base composition skew values were computed as follows: AT skew = (A − T)/(A + T) and GC skew = (G − C)/(G + C) [25].

### 2.4. Phylogenetic Analysis

The phylogenetic analysis included a total of 31 species, comprising 19 species from the Coccomorpha, 4 species from the Aphidomorpha, 3 species from the Aleyrodomorpha, 2 species from the Psyllomorpha, and 3 species of order Thysanoptera. Phylogenetic relationship was inferred using Maximum Likelihood (ML) and Bayesian Inference (BI). A dataset comprising protein-coding genes from 31 species was concatenated in Phylosuite v1.2.2 for phylogenetic analysis [26]. Thirteen sequences were aligned in batches with MAFFT v7.505 using the ‘G-INS-i (accurate)’ strategy and codon alignment mode [27]. Ambiguously aligned fragments of 13 alignments were removed in batches using Gblocks v0.91b [28]. Finally, the 13 protein-coding genes were concatenated in Phylosuite. Before constructing Maximum Likelihood tree and Bayesian Inference tree, the protein-coding dataset was partitioned schemes for evolutionary models were selected using PartitionFinder v2.1.1 [29]. For the Maximum Likelihood tree, the best partitioning scheme and evolutionary models for the 13 protein-coding genes were selected using the greedy algorithm combined with the AICc criterion. However, for the Bayesian tree, the greedy algorithm was applied in conjunction with the BIC criterion. Maximum likelihood phylogenies were inferred using IQ-TREE v2.2.0 under the edge-linked partition model for 5000 ultrafast bootstraps, as well as the Shimodaira–Hasegawa-like approximate likelihood ratio test [30,31,32]. Bayesian Inference phylogenies were inferred using MrBayes v3.2.6 under the partition model (2 parallel runs, 2,000,000 generations) [33], in which the initial 25% of sampled data were discarded as burn-in.

## 3. Results

### 3.1. General Features and Nucleotide Composition

**Figure 1:**
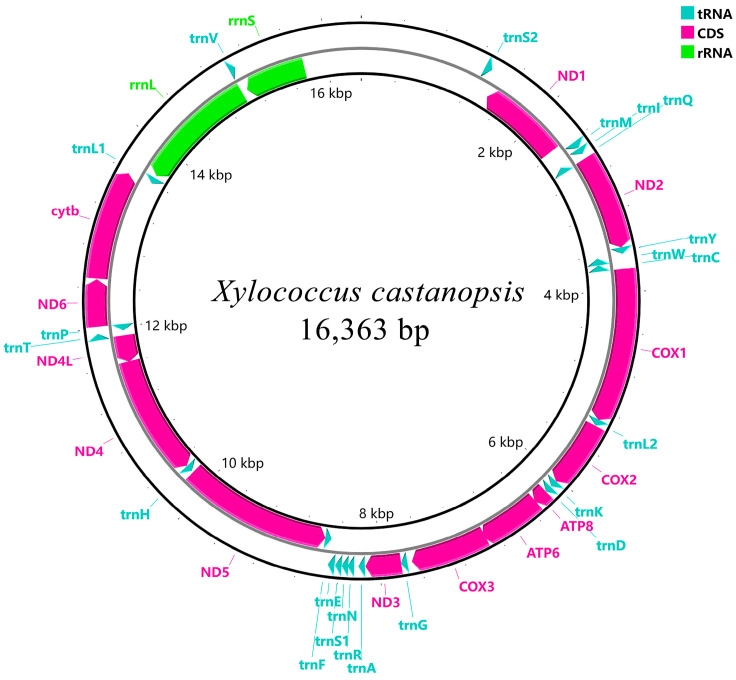
Circular maps of the mitogenomes of *X. castanopsis*.

The length of the mitogenome sequence of *X. castanopsis* was 16,363 bp, containing 13 PCGs, 22 tRNAs and 2 rRNAs, amongst which 9 PCGs and 15 tRNAs were encoded on the forward strand (+) and 13 genes were encoded by the reverse strand (-) (Figure 1 and Table 2). Ten regions of gene overlap between adjacent genes were detected in the mitogenome, ranging from −1 to −14 bp, and 20 intergenic regions, ranging from 1 to 94 bp. The overall base composition of the mitogenome sequence was 42.9% T, 46.3% A, 7.7% C and 3.1% G, with a strong bias towards A + T (89.2%) (Table 3).

### 3.2. Protein-Coding Genes

**Figure 2:**
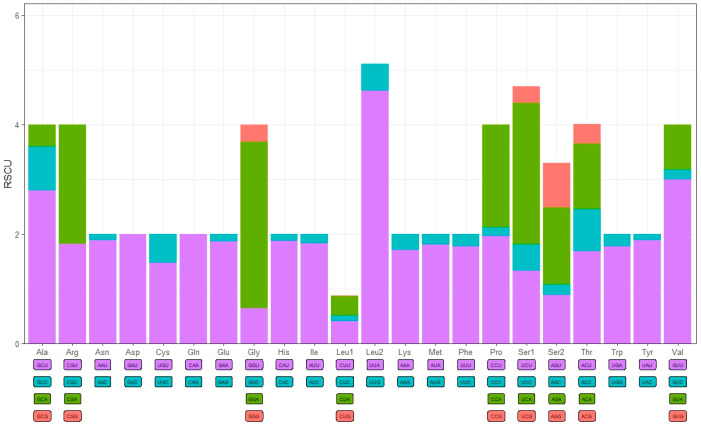
The relative synonymous codon usage (RSCU) in the mitogenome of *X. castanopsis*.

The 13 protein-coding genes (PCGs) span a total of 10,747 bp, comprising 65.7% of the complete mitogenome length of *X*. *castanopsis*, with a high A + T content of 88.8% (Table 3). The AT skew values for the 13 PCGs range from −0.20 in *ND4* to 0.12 in *ATP8*, while the GC skew values vary from −0.78 in *ATP8* to 0.73 in *ND4L*. The start codons for all protein-coding genes were ATN, while the stop codons varied: *COX2* ended with T-, *ND5* ended with TAG, and the remaining genes all terminated with TAA. The relative synonymous codon usage (RSCU) was depicted in Figure 2. The usage of codons for protein-coding genes is presented in descending order of frequency as follows: AAT, ATT, TTT, TAT and ATA (Table 4).

### 3.3. tRNA Genes and rRNA Genes

**Figure 3:**
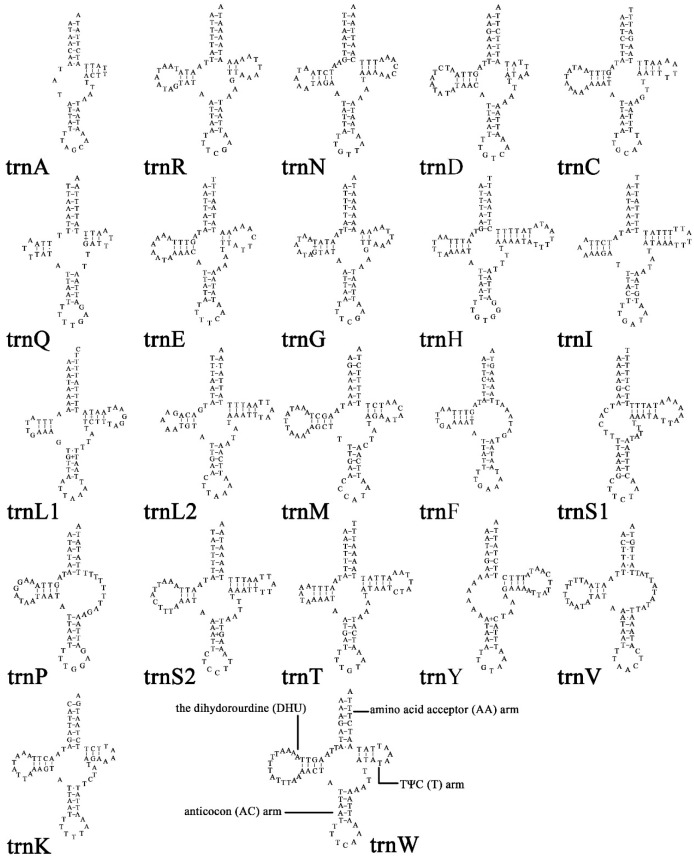
Secondary structures of the transfer RNA genes (tRNAs) in *X. castanopsis* mitogenome.

The tRNAs of *X. castanopsis* had a length ranging from 47 bp (*trnA*) to 73 bp (*trnH*). Among 22 tRNAs, 16 tRNAs (*trnC*, *trnD*, *trnE*, *trnG*, *trnH*, *trnI*, *trnK*, *trnL1*, *trnL2*, *trnM*, *trnN*, *trnQ*, *trnR*, *trnS2*, *trnT*, and *trnW*) exhibited a complete cloverleaf structure, while 3 tRNAs (*trnA*, *trnS1*, and *trnY*) lacked the dihydrouridine (DHU) arm, and 3 other tRNAs (*trnF*, *trnP*, and *trnV*) lacked the TψC (T) arm (Figure 3).

The two rRNAs were both on the reverse strand in the mitogenome of *X. castanopsis*. The *rrnL* gene was 1,340 bp with 90.1% A + T content. The *rrnS* gene was 668 bp with 88.6% A + T content.

### 3.4. Gene Rearrangements Analysis

**Figure 4:**
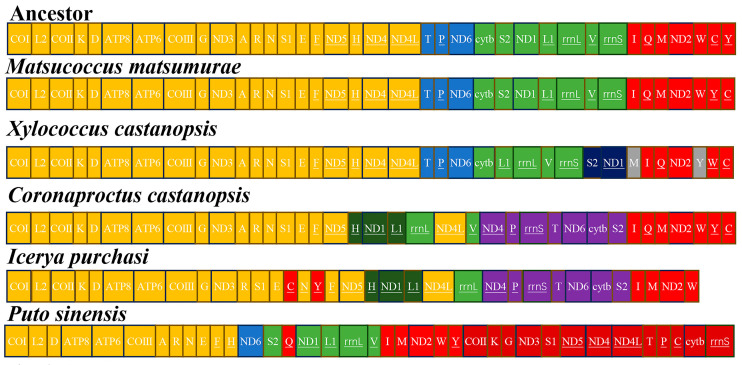
Gene orders in ancestor and scale insects’ mitogenomes. The order was divided into four relatively conserved gene regions colored with yellow, blue, green and red.

The mitochondrial genome of *X. castanopsis* exhibits significant gene rearrangements when compared to the ancestral sequence and other archeaococcoids (Figure 4). The genes *trnS2-ND1* and *trnL1-rrnL-trnV-rrnS* have undergone transposition (T), and the positions of *trnI-trnQ* and *trnM* have been transposed (T). Additionally, the gene *trnV* has undergone inversion (I), and the rearrangement involving *trnY* and *trnW-trnC* is an inverted transposition (iT).

### 3.5. Phylogenetic Analysis of Coccomorpha

**Figure 5:**
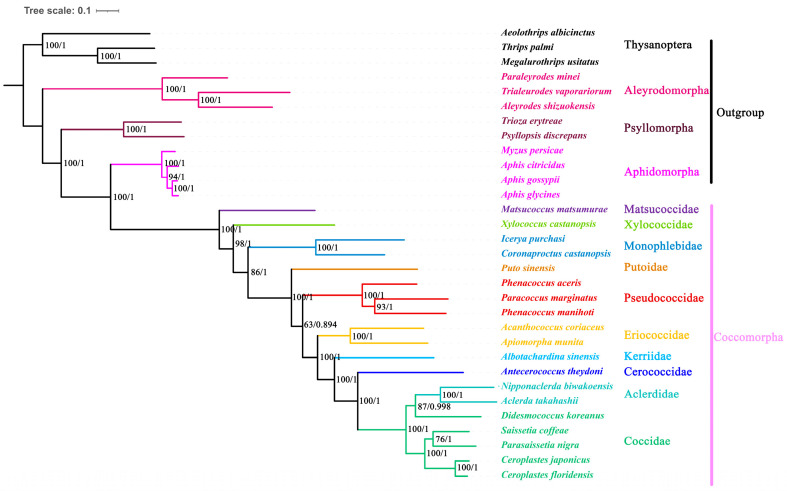
Phylogenetic trees inferred from mitogenomes of Coccomorpha under ML and BI. The numbers at nodes indicate Maximum Likelihood bootstrap values (**left**) and Bayesian posterior probabilities (**right**).

The phylogenetic trees reconstructed using the Maximum Likelihood and Bayesian Inference methods, based on the dataset of 13 protein-coding genes, were identical and had high support (Figure 5). Four infraorders (Aleyrodomorpha, Psyllomorpha, Aphidomorpha and Coccomorpha) were recovered as monophyletic with strong support. Aleyrodomorpha was sister to other infraorders. The phylogenetic relationship of Aleyrodomorpha + (Psyllomorpha + (Aphidomorpha + Coccomorpha))) was found with high statistical support (bootstrap value = 100%, posterior possibility = 1).

Within Coccomorpha, the Matsucoccidae was a sister to other coccoid families analyzed here. Xylococcidae formed a sister group with all other scale insects excluding Matsucoccidae. The two species of Monophlebidae formed separate clades, strongly supporting their monophyly (bootstrap value = 98%, posterior possibility = 1). Putoidae was found to be the sister group to all neococcids. The Pseudococcidae was grouped with the remaining neococcids. Among the remaining neococcids, the phylogenetic relationship of (Eriococcidae + (Kerriidae + (Cerococcidae + (Aclerdidae + Coccidae)))) was found with high statistical support (bootstrap value = 100%, posterior possibility = 1).

## 4. Discussion

Rearrangements in mitochondrial genomes are common in scale insects [14,15,16,17,18]. The Matsucoccidae exhibits gene arrangements that are similar to the ancestral sequence, with only individual tRNA transpositions (T). However, due to morphological similarities between xylococcids and matsucoccids, they are often placed by cocciologists as tribe Xylococcini and Matsucoccini within Margarodidae sensu lato, or alternatively [5,34], genera such as *Xylococcus* and *Matsucoccus* are classified under Xylococcidae [6]. Our results show that, compared to the ancestral arrangement, Matsucoccidae exhibits tRNA transpositions, while Xylococcidae exhibits more rearrangements, such as tRNA transpositions (T), inversions (I), inverted transpositions (iT), and transpositions (T) between the gene clusters *trnS2-ND1* and *trnL1-rrnL-trnV-rrnS*.

The Coccomorpha is divided into two informal groups: archaeococcids and neococcoids. Archaeococcids are characterized by the presence of abdominal spiracles, and adult males possess compound eyes or a row of unicorneal eyes encircling the head, while the neococcoids lack the abdominal spiracles and compound eyes (except for Kermesidae and some Coccidae) [4,35]. Within Coccomorpha, Matsucoccidae is considered to be more morphologically similar to *Orthezia* and *Aphis* (Aphidomorpha) than to Xylococcidae [36]. However, Koteja proposed a potential relationship between Matsucoccidae and Xylococcidae based on mouthpart morphology, while suggesting a less likely affinity with Ortheziidae [7]. Due to the lack of data on Ortheziidae, our results recognize Matsucoccidae as the sister with other scale insects, showing closer phylogenetic proximity to Xylococcidae, consistent with previous findings [34,37,38].

Although the Xylococcidae and Matsucoccidae were included in Margarodidae sensu lato, the morphology of adult males and females of Xylococcidae is distinct from that of Matsucoccidae. The pores are present in the atrium of the abdominal spiracles of Xylococcidae, while absent in those of Matsucoccidae. Additionally, bitubular ducts are absent in Xylococcidae but present in Matsucoccidae [39]. In males, Matsucoccidae possess only a cluster of cupolae at the wing base, whereas Xylococcidae exhibit both a row of cupolae along the subcostal complex and a cluster at the wing base [8]. Morphological analysis of adult males reveals close phylogenetic relationships between Matsucoccidae and Xylococcidae, evidenced by shared derived characteristics including (1) the presence of a dorsal waxy tuft, (2) a group of very small setae on the dorsal surface of antennal pedicel and coxal bases, and (3) the development of alar folds [40,41]. In our study, Xylococcidae was grouped with all other scale insects except for Matsucoccidae, with strong support (98% bootstrap support and one posterior probability). This mainly supports the phylogenetic result obtained by Hodgson and Hardy based on male morphological characteristics [38]. Monophlebidae has consistently shown strong monophyly, whether based on molecular or morphological data [37,42,43]. However, there is still no satisfactory classification system [44]. Our results suggest that Monophlebidae forms a clade. It forms a sister with all other scale insects.

## Figures and Tables

**Table 1:** Species involved in the phylogenetic analysis.

Order	Family	Species	NCBI Accession Number
Thysanoptera	Aeolothripidae	*Aeolothrips albicinctus*	PP932666
	Thripidae	*Thrips palmi*	MN127983
		*Megalurothrips usitatus*	ON815612
Hemiptera	Aleyrodidae	*Aleyrodes shizuokensis*	MT880225
		*Paraleyrodes minei*	PP727237
		*Trialeurodes vaporariorum*	AY521265
	Psyllidae	*Psyllopsis discrepans*	OR608273
		*Trioza erytreae*	MG989238
	Aphididae	*Aphis citricidus*	MK540501
		*Aphis glycines*	MK111111
		*Aphis gossypii*	OR449278
		*Myzus persicae*	KU236024
	Matsucoccidae	*Matsucoccus matsumurae*	PP103290
	Monophlebidae	*Icerya purchasi*	OX7316821
		*Coronaproctus castanopsis*	SRR26067561
	Putoidae	*Puto sinensis*	PQ727056
	Xylococcidae	*Xylococcus castanopsis*	PV227316
	Pseudococcidae	*Paracoccus marginatus*	PP060471
		*Phenacoccus aceris*	SRR14087799
		*Phenacoccus manihoti*	MZ958983
	Eriococcidae	*Acanthococcus coriaceus*	OP351525
		*Apiomorpha munita*	OP351523
	Kerriidae	*Albotachardina sinensis*	OP351521
	Cerococcidae	*Antecerococcus theydoni*	OP351522
	Aclerdidae	*Nipponaclerda biwakoensis*	OP351526
		*Aclerda takahashii*	MW839575
	Coccidae	*Diensmococcus koreanus*	MW302211
		*Saissetia coffeae*	MN863803
		*Parasaissetia nigra*	OK040656
		*Ceroplastes japonicus*	OP351524
		*Ceroplastes floridensis*	OK040657

**Table 2:** Mitochondrial genome characteristics of *X. castanopsis*.

Gene	Location	Size	Strand	Start Codon	Stop Codon	Anticodon	Intergenic Length
*trnS2*	1273–1344	72	+	-	-	CCT	-
*ND1*	1439–2356	918	-	ATA	TAA	-	94
*trnM*	2424–2493	70	+	-	-	CAT	67
*trnI*	2500–2565	66	+	-	-	GAT	6
*trnQ*	2564–2617	54	-	-	-	TTG	−2
*ND2*	2617–3564	948	+	ATT	TAA	-	−1
*trnY*	3565–3625	61	+	-	-	GTA	0
*trnW*	3631–3703	61	-	-	-	TCA	5
*trnC*	3705–3773	69	-	-	-	GCA	1
*COX1*	3775–5304	1530	+	ATG	TAA	-	1
*trnL2*	5300–5365	66	+	-	-	TAA	−5
*COX2*	5369–6038	670	+	ATA	T-	-	3
*trnK*	6039–6104	66	+	-	-	TTT	0
*trnD*	6114–6178	65	+	-	-	GTC	9
*ATP8*	6179–6328	150	+	ATA	TAA	-	0
*ATP6*	6318–6947	630	+	ATA	TAA	-	−11
*COX3*	6937–7683	750	+	ATG	TAA	-	−14
*trnG*	7729–7792	64	+	-	-	TCC	45
*ND3*	7790–8143	354	+	ATA	TAA	-	−3
*trnA*	8157–8203	47	+	-	-	AGC	13
*trnR*	8259–8312	54	+	-	-	TCG	55
*trnN*	8312–8375	64	+	-	-	GTT	−1
*trnS1*	8377–8439	60	+	-	-	TCT	1
*trnE*	8447–8512	66	+	-	-	TTC	7
*trnF*	8512–8568	57	-	-	-	GAA	−1
*ND5*	8574–10,205	1632	-	ATT	TAG	-	5
*trnH*	10,227–10,299	73	-	-	-	GTG	21
*ND4*	10,303–11,610	1308	-	ATG	TAA	-	3
*ND4L*	11,604–11,897	294	-	ATT	TAA	-	−7
*trnT*	11,887–11,955	69	+	-	-	TGT	−11
*trnP*	11,959–12,017	59	-	-	-	TGG	3
*ND6*	12,021–12,485	465	+	ATA	TAA	-	3
*cytb*	12,493–13,579	1086	+	ATA	TAA	-	7
*trnL1*	13,603–13,669	67	-	-	-	TAA	24
*rrnL*	13,700–15,009	1340	-	-	-	-	0
*trnV*	15,010–15,071	62	+	-	-	AAC	0
*rrnS*	15,072–15,739	668	-	-	-	-	0

“+/-” indicates whether the gene is located on the forward or reverse strand, respectively. The minus signs (-) appearing in the “Start codon”, “Stop codon”, and “Anticodon” columns simply denote blank entries or absence of relevant data for those particular fields.

**Table 3:** The nucleotide composition of the mitochondrial genome.

Gene	% T	% C	% A	% G	% A + T	AT Skew	GC Skew
*ATP6*	47.0	7.5	43.0	2.5	90.0	−0.04	−0.49
*ATP8*	41.3	5.3	52.7	0.7	94.0	0.12	−0.78
*COX1*	43.9	10.5	38.6	7.1	82.5	−0.06	−0.19
*COX2*	42.4	8.1	45.2	4.3	87.6	0.03	−0.31
*COX3*	49.3	6.5	41.2	3.0	90.4	−0.09	−0.37
*cytb*	45.3	8.9	41.0	4.8	86.3	−0.05	−0.30
*ND1*	51.7	3.3	36.3	8.7	88.0	−0.18	0.45
*ND2*	49.4	6.8	42.8	1.0	92.2	−0.07	−0.74
*ND3*	44.6	5.4	46.9	3.1	91.5	0.02	−0.27
*ND4*	54.0	3.0	35.7	7.3	89.7	−0.20	0.42
*ND4L*	53.7	1.0	38.8	6.5	92.5	−0.16	0.73
*ND5*	53.7	3.0	36.4	6.9	90.1	−0.19	0.39
*ND6*	48.8	4.7	45.2	1.3	94.0	−0.04	−0.57
PCGs	48.8	6.0	40.0	5.2	88.8	−0.10	−0.07
*rrnL*	48.1	2.6	42.1	7.2	90.1	−0.07	0.47
*rrnS*	45.1	3.1	43.6	8.2	88.6	−0.02	0.45
2 rRNA	47.0	2.8	42.6	7.6	89.6	−0.05	0.46
22 tRNA	44.3	4.8	45.9	5.0	90.2	0.02	0.01
Total	42.9	7.7	46.3	3.1	89.2	0.04	−0.43

**Table 4:** The utilization of codons of the mitogenome of *X. castanopsis*.

Amino Acid	Codon	Count	RSCU	Amino Acid	Codon	Count	RSCU
Phe	UUU(F)	447	1.77	Ser	UCU(S)	36	1.33
	UUC(F)	58	0.23		UCC(S)	13	0.48
Leu	UUA(L)	235	4.62		UCA(S)	70	2.59
	UUG(L)	25	0.49		UCG(S)	8	0.3
Leu (c)	CUU(L)	21	0.41	Ser (s)	AGU(S)	24	0.89
	CUC(L)	5	0.1		AGC(S)	5	0.19
	CUA(L)	18	0.35		AGA(S)	38	1.41
	CUG(L)	1	0.02		AGG(S)	22	0.81
Ile	AUU(I)	478	1.83	Thr	ACU(T)	38	1.69
	AUC(I)	44	0.17		ACC(T)	17	0.76
Met	AUA(M)	243	1.81		ACA(T)	27	1.2
	AUG(M)	25	0.19		ACG(T)	8	0.36
Val	GUU(V)	18	3	Ala	GCU(A)	7	2.8
	GUC(V)	1	0.17		GCC(A)	2	0.8
	GUA(V)	5	0.83		GCA(A)	1	0.4
	GUG(V)	0	0		GCG(A)	0	0
Tyr	UAU(Y)	370	1.89	Cys	UGU(C)	14	1.47
	UAC(Y)	22	0.11		UGC(C)	5	0.53
*	UAA(*)	170	1.68	Trp	UGA(W)	68	1.77
	UAG(*)	32	0.32		UGG(W)	9	0.23
His	CAU(H)	31	1.88	Arg	CGU(R)	5	1.82
	CAC(H)	2	0.12		CGC(R)	0	0
Gln	CAA(Q)	23	2		CGA(R)	6	2.18
	CAG(Q)	0	0		CGG(R)	0	0
Asn	AAU(N)	483	1.89	Pro	CCU(P)	24	1.96
	AAC(N)	29	0.11		CCC(P)	2	0.16
Lys	AAA(K)	177	1.71		CCA(P)	23	1.88
	AAG(K)	30	0.29		CCG(P)	0	0
Asp	GAU(D)	38	2	Gly	GGU(G)	8	0.64
	GAC(D)	0	0		GGC(G)	0	0
Glu	GAA(E)	27	1.86		GGA(G)	38	3.04
	GAG(E)	2	0.14		GGG(G)	4	0.32

The “*” denotes stop codon.

## Data Availability

The data that support the findings of this study are openly available in the National Center for Biotechnology Information at https://www.ncbi.nlm.nih.gov/nuccore (accessed on 6 March 2025), reference number PV227316.

## References

[1] Gullan P.J., Martin J.H., Resh V.H., Cardé R.T. (2009). Sternorrhyncha: (Jumping plant-lice, whiteflies, aphids, and scale insects). Encyclopedia of Insects.

[2] García Morales M., Denno B.D., Miller D.R., Miller G.L., Ben-Dov Y., Hardy N.B. ScaleNet: A Literature-Based Model of Scale Insect Biology and Systematics. Database. http://scalenet.info.

[3] Borchsenius N.S. (1958). On the evolution and phylogenetic interrelations of Coccoidea (Insecta, Homoptera). Zool. Zhurnal.

[4] Williams D.J., Gullan P.J., Miller D.R., Matile-Ferrero D., Han S.I. (2011). A study of the scale insect genera *Puto* ignoret (Hemiptera: Sternorrhyncha: Coccoidea: Putoidae) and *Ceroputo* Sulc (Pseudococcidae) with a comparison to *Phenacoccus* Cockerell (Pseudococcidae). Zootaxa.

[5] Morrison H. (1928). A classification of the higher groups and genera of the coccid family Margarodidae. USDA Tech. Bull..

[6] Zahradnik J. (1959). Cervci—Coccinea. Scale insects—Coccinea. Bestimmungstabellen Tschechoslow. Fauna.

[7] Koteja J. (1974). On the phylogeny and classification of the scale insects (Homoptera, Coccinea) (discussion based on the morphology of the mouthparts). Acta Zool. Cracoviensia.

[8] Koteja J. (1996). Scale insects (Homoptera: Coccinea) a day after. Studies on Hemipteran Phylogeny.

[9] Wu S.A., Huang S.B., Dong Q.-G. (2017). First records of the family Xylococcidae (Hemiptera: Coccomorpha) in China, with description of a new species. Zootaxa.

[10] Ben-Dov Y. (2011). An updated checklist of the scale insects (Hemiptera: Coccoidea) of the Margarodidae *sensu lato* group. Zootaxa.

[11] Nass M.M., Nass S. (1963). Intramitochondrial fibers with DNA characteristics: I. Fixation and electron staining reactions. J. Cell Biol..

[12] Beard C.B., Hamm D.M., Collins F.H. (1993). The mitochondrial genome of the mosquito *Anopheles gambiae*: DNA sequence, genome organization, and comparisons with mitochondrial sequences of other insects. Insect Mol. Biol..

[13] Manchekar M., Scissum-Gunn K., Song D., Khazi F., McLean S.L., Nielsen B.L. (2006). DNA recombination activity in soybean mitochondria. J. Mol. Biol..

[14] Hu K., Yu S., Zhang N., Tian M., Ban Q., Fan Z., Qiu J. (2022). The first complete mitochondrial genome of Matsucoccidae (Hemiptera, Coccoidea) and implications for its phylogenetic position. Biodivers. Data J..

[15] Hou Y.F., Wei J.F., Zhao T.Y., Li C.F., Wang F. (2023). First complete mitochondrial genome of the tribe Coccini (Hemiptera, Coccomorpha, Coccidae) and its phylogenetic implications. ZooKeys.

[16] Lu C.C., Huang X., Deng J. (2023). Mitochondrial genomes of soft scales (Hemiptera: Coccidae): Features, structures and significance. BMC Genom..

[17] Xu H., Liu X., Wang P., Li H., Wu S.A. (2023). Phylogenetic Implications of Mitogenomic Sequences and Gene Rearrangements of Scale Insects (Hemiptera and Coccoidea). Insects.

[18] Deng J., Lu C.C., Huang X.L. (2019). The first mitochondrial genome of scale insects (Hemiptera: Coccoidea). Mitochondrial DNA B Resour..

[19] Dierckxsens N., Mardulyn P., Smits G. (2017). NOVOPlasty: De novo assembly of organelle genomes from whole genome data. Nucleic Acids Res..

[20] Donath A., Jühling F., Al-Arab M., Bernhart S.H., Reinhardt F., Stadler P.F., Middendorf M., Bernt M. (2019). Improved Annotation of Protein-Coding Genes Boundaries in Metazoan Mitochondrial Genomes. Nucleic Acids Res..

[21] Kumar S., Stecher G., Tamura K. (2016). MEGA7: Molecular Evolutionary Genetics Analysis version 7.0 for bigger datasets. Mol. Biol. Evol..

[22] Laslett D., Canback B. (2008). ARWEN: A program to detect tRNA genes in metazoan mitochondrial nucleotide sequences. Bioinformatics.

[23] Wickham H. (2016). Ggplot2: Elegant Graphics for Data Analysis.

[24] R Core Team (2014). R: A Language and Environment for Statistical Computing.

[25] Perna N.T., Kocher T.D. (1995). Patterns of nucleotide composition at fourfold degenerate sites of animal mitochondrial genomes. J. Mol. Evol..

[26] Zhang D., Gao F., Jakovlić I., Zou H., Zhang J., Li W.X., Wang G.T. (2020). PhyloSuite: An integrated and scalable desktop platform for streamlined molecular sequence data management and evolutionary phylogenetics studies. Mol. Ecol. Resour..

[27] Katoh K., Standley D.M. (2013). MAFFT multiple sequence alignment software version 7: Improvements in performance and usability. Mol. Biol. Evol..

[28] Talavera G., Castresana J. (2007). Improvement of phylogenies after removing divergent and ambiguously aligned blocks from protein sequence alignments. Syst. Biol..

[29] Lanfear R., Frandsen P.B., Wright A.M., Senfeld T., Calcott B. (2017). PartitionFinder 2: New methods for selecting partitioned models of evolution for molecular and morphological phylogenetic analyses. Mol. Biol. Evol..

[30] Nguyen L.T., Schmidt H.A., von Haeseler A., Minh B.Q. (2015). IQ-TREE: A fast and effective stochastic algorithm for estimating maximum-likelihood phylogenies. Mol. Biol. Evol..

[31] Minh B.Q., Nguyen M.A., von Haeseler A. (2013). Ultrafast approximation for phylogenetic bootstrap. Mol. Biol. Evol..

[32] Guindon S., Dufayard J.F., Lefort V., Anisimova M., Hordijk W., Gascuel O. (2010). New algorithms and methods to estimate maximum-likelihood phylogenies: Assessing the performance of PhyML 3.0. Syst. Biol..

[33] Ronquist F., Teslenko M., van der Mark P., Ayres D.L., Darling A., Höhna S., Larget B., Liu L., Suchard M.A., Huelsenbeck J.P. (2012). MrBayes 3.2: Efficient Bayesian phylogenetic inference and model choice across a large model space. Syst. Biol..

[34] Gavrilov-Zimin I.A. (2018). Ontogenesis, morphology and higher classification of archaeocococcids (Homoptera: Coccinea: Orthezoiidea). Zoosyst. Ross..

[35] Hodgson C.J. (2020). A review of neococcid scale insects (Hemiptera: Sternorrhyncha: Coccomorpha) based on the morphology of the adult males. Zootaxa.

[36] Beardsley J.W. (1968). External morphology of the adult male of *Matsucoccus bisetosus*. Ann. Entomol. Soc. Am..

[37] Hodgson C.J., Hardy N.B. (2013). The phylogeny of the superfamily Coccoidea (Hemiptera: Sternorrhyncha) based on the morphology of extant and extinct macropterous males. Syst. Entomol..

[38] Vea I.M., Grimaldi D.A. (2015). Diverse new scale insects (Hemiptera: Coccoidea) in amber from the Cretaceous and Eocene with a phylogenetic framework for fossil Coccoidea. Am. Mus. Novit..

[39] Kondo T., Watson G.W. (2022). Encyclopedia of Scale Insect Pests. Encyclopedia of Scale Insect Pests.

[40] Hodgson C.J., Foldi I. (2006). A review of the *Margarodidae sensu* Morrison (Hemiptera: Coccoidea) and some related taxa based on the morphology of adult males. Zootaxa.

[41] Koteja J. (2008). Xylococcidae and related groups (Hemiptera: Coccinea) from Baltic amber. Pr. Muz. Ziemi.

[42] Gullan P.J., Cook L.G. (2007). Phylogeny and higher classification of the scale insects (Hemiptera: Sternorrhyncha: Coccoidea). Zootaxa.

[43] Song N., Wang M., Tang H., Dang Z. (2024). A phylogenetic analysis of scale insects (Hemiptera, Coccoidea) based on genomic and transcriptomic data. J. Syst. Evol..

[44] Li J.N., Xu H., Wu S.A. (2023). A new genus and species of giant mealybugs (Hemiptera: Coccomorpha: Monophlebidae) from eastern China. Zootaxa.

